# Estrogen Induces Vav1 Expression in Human Breast Cancer Cells

**DOI:** 10.1371/journal.pone.0099052

**Published:** 2014-06-06

**Authors:** Ming-juan Du, Xiang-dong Chen, Xiao-li Zhou, Ya-juan Wan, Bei Lan, Cui-zhu Zhang, Youjia Cao

**Affiliations:** 1 Key Laboratory of Microbial Functional Genomics of Ministry of Education, College of Life Sciences, Nankai University, Tianjin, P. R. China; 2 State Key Laboratory of Medicinal Chemical Biology, College of Life Sciences, Nankai University, Tianjin, P. R. China; Sun Yat-sen University Medical School, China

## Abstract

Vav1, a guanine nucleotide exchange factor (GEF) for Rho family GTPases, is a hematopoietic protein involved in a variety of cellular events. In recent years, aberrant expression of Vav1 has been reported in non-hematopoietic cancers including human breast cancer. It remains to be answered how Vav1 is expressed and what Vav1 does in its non-resident tissues. In this study, we aimed to explore the mechanism for Vav1 expression in breast cancer cells in correlation with estrogen-ER pathway. We not only verified the ectopic expression of Vav1 in human breast cancer cell lines, but also observed that Vav1 expression was induced by 17β-estradiol (E_2_), a typical estrogen receptor (ER) ligand, in ER-positive cell lines. On the other hand, Tamoxifen, a selective estrogen receptor modulator (SERM), and ICI 182,780, an ER antagonist, suppressed the expression of Vav1. The estrogen receptor modulating Vav1 expression was identified to be α form, not β. Furthermore, treatment of E_2_ increased the transcription of *vav*1 gene by enhancing the promoter activity, though there was no recognizable estrogen response element (ERE). Nevertheless, two regions at the *vav*1 gene promoter were defined to be responsible for E_2_-induced activation of *vav*1 promoter. Chromatin immunoprecipitation (ChIP) and co-immunoprecipitation (Co-IP) analyses suggested that ERα might access to the *vav*1 promoter via interacting with transcription factors, c-Myb and ELF-1. Consequently, the enhanced expression of Vav1 led to the elevation of Cyclin D1 and the progression of cell cycle. The present study implies that estrogen-ER modulates the transcription and expression of Vav1, which may contribute to the proliferation of cancerous cells.

## Introduction

Breast cancer is the most common death-causing cancer in females [1]. The persistent exposure to estrogen has been observed to closely correlate with the development of breast cancer [2]–[5]. The expression and responsiveness of estrogen receptor (ER) has been applied as one of the most important markers for the breast cancer classification and prognosis [6]. Two forms of estrogen receptors, ERα and ERβ, have been identified [7]. ERα is the dominant form in the breast and uterus, whereas ERβ has a wider distribution profile that expands in tissues such as prostate, ovary, lung, and spleen [8]. As a ligand, estrogen binds to ER, and induces its conformational change to activate it. The activated ER associates with the estrogen response element (ERE) at the promoter regions of a variety of genes [9], or complexes with other transcription factors, such as AP1 [10], SP1 [11], or E2F1 [12], modulating the expression of target genes that are involved in cell cycle checkpoint [12], [13], cell proliferation [14], [15], and apoptosis [16].

Vav1 is first identified as a proto-oncogene in hematopoietic cells [17], with the renowned character as a guanine nucleotide exchange factor (GEF) for RhoGTPases. A plethora of studies revealed that Vav1 is a multidomain protein which not only activates RhoGTPases for cytoskeleton reorganization during lymphocytes activation [18], but also plays a GEF-independent role in diverse cellular processes including calcium mobilization in T cells [19]. The oncogenic form, lacking the N-terminal Calponin homology (CH) domain, is obtained by its transforming effect on NIH3T3 fibroblast cells [20]. Meanwhile, evidence unveils the non-hematopoietic expression profile of Vav1, which associates with several human tumor malignancies, such as neuroblastoma [21], lung cancer [22], and pancreatic ductal adenocarcinomas [23]. Knocking down of *vav*1 gene in lung cancer and pancreatic cells leads to the decreased cell proliferation and reduces tumor size in nude mice [22], [23]. In addition, patients with Vav1-positive pancreatic tumors exhibit poorer prognosis and lower survival rate than those with Vav1-negative tumors [23], suggesting that the ectopic expression of Vav1 plays an innegligible role in tumor development and progression.

Recently, the aberrant expression of Vav1 has been reported and its correlation with estrogen receptor has been addressed in human breast cancer [24]–[27]. Herein we aim to investigate the modulation of Vav1 expression in breast cancer cells, and the effect of Vav1 on breast cancer cell proliferation. By confirming the increased *vav*1 mRNA and protein in several ER-positive cell lines, we found that the transcription and expression of Vav1 was significantly enhanced by E_2_ treatment in a time- and dose-dependent manner via ERα. We further addressed that E_2_-induced *vav*1 transcription involved the complex of ERα with other transcription factors. Finally, we showed that the amount of Vav1 expression correlated with the expression of Cyclin D1 and influenced the cell cycle progression in breast cancer cells. Our data suggested that estrogen may promote breast cancer cell growth partially by triggering the aberrant expression of Vav1.

## Methods and Materials

### Antibodies and reagents

The anti-ERα (sc-73479), anti-ERβ (sc-373853), anti-Vav1 (sc-132), anti-c-Myb (sc-517 X), and anti-ELF-1 (sc-631X) antibodies and normal IgG (rabbit, sc-2027; mouse, sc-2025) were purchased from Santa Cruz Biotechnology (CA, USA). The anti-Cyclin D1 antibody (BA0770) was purchased from Boster (Wuhan, China). ICI 182,780 (Fulvestrant), Tamoxifen, 17β-estradiol (E_2_), Dimethyl sulfoxide (DMSO), 1,3,5-Tris(4-hydroxyphenyl)-4-propyl-1H-pyrazole (PPT), 2,3-Bis(4-hydroxyphenyl) propionitrile (DPN), and anti-α-tubulin antibody were purchased from Sigma (MO, USA).

### Cell lines and culture

Human breast cancer cell lines (ER positive: MCF7 and T47D; ER negative: MDA-MB-231 and MDA-MB-157) were originally from American Type Culture Collection (ATCC) and maintained in phenol free RPMI 1640 medium supplemented with 10% fetal bovine serum (FBS) (GIBCO, NY, USA). For MCF7 and T47D cells, 0.01 mg/ml human recombinant insulin was added to the medium. The immortalized breast epithelial line 184A1 was maintained in MEGM (Cambrex, NJ, USA) that consisted of modified MCDB 170 medium supplemented with ∼52 µg/mL bovine pituitary extract, 10 ng/mL epidermal growth factor, 0.5 µg/mL hydrocortisone, 5 µg/mL insulin, 50 µg/mL gentamicin sulfate, and 50 ng/mL amphotericin B. Jurkat T leukemia cells and *vav*1-null Jurkat T cells (J.Vav1) were carried in lab as described previously [28] and were grown in RPMI 1640 medium containing 10% FBS. HEK293T cell line was obtained from ATCC, and grown in DMEM supplemented with 10% FBS.

### RNA isolation and reverse transcription

MCF7 and T47D cells were cultured in phenol free RPMI 1640 medium for 24 h to deprive estrogen, and then treated with 10^−7^ mol/L of E_2_ for 24 h. The mRNA was extracted from the cells using PolyATract System 1000 (Promega, WI, USA) and reverse-transcribed with Reverse Transcription System (Promega, WI, USA).

### Quantitative Real-time PCR

The cDNAs isolated from cell lines were used as templates. Expression of *vav*1 mRNA was determined by qRT-PCR with EvaGreen Dye (Biotium, CA, USA) using Real-time PCR System (Bio-Rad, CA, USA). The relative gene expression was calculated by 2^−ΔΔCt^ method [29] and normalized to housekeeping gene hypoxanthine phosphoribosyltransferase 1 (HPRT). The sequences of the PCR primers were designed as follows: *vav*1: sense: 5'-TACGGGCTTCCTCCACCCCC-3', antisense: 5'-TGGGGCCTGCGTACCAGAGA-3'; HPRT: sense: 5'-TGACACTGGCAAAACAATGCA-3', antisense: 5'-GGTCCTTTTCACCAGCAAGCT-3'.

### Luciferase reporter assay

The *vav*1 promoter constructs were obtained as described [30]. MCF7 cells were transfected with 2 µg of total plasmid DNA containing *Renilla* luciferase vector pRL-TK (Promega, WI, USA) and *vav*1 reporter construct, pVav1-Luc, per 1×10^5^ cells by Lipofectamine 2000 (Invitrogen, CA, USA). Cells were cultured in RPMI 1640 medium for 24 h followed by medium containing E_2_ (10^−7^ mol/L) for 48 h, then lysed for luciferase activity analyses with Dual Luciferase Assay kit (Promega, WI, USA) and TD20/20 luminometer (Turner Designs Inc, CA, USA). The promoter activity was presented as the ratio of the firefly luciferase activity to *Renilla* luciferase activity. To determine the effect of ERs on the promoter activity of *vav*1, MCF7 cells transfected with pVav1-Luc were pre-treated with Tamoxifen or ICI 182,780 for 30 min in prior to E_2_ treatment or treated with ER type-specific agonists, PPT or DPN. The cells were then harvested for luciferase assay as described above.

### Chromatin immunoprecipitation (ChIP) assay

ChIP assay was carried out as previously described [31]. Briefly, T47D cells were pre-cultured in serum-free medium and then treated with DMSO or E_2_ (10^−7^ mol/L) for 4 h or pre-treated with Tamoxifen (10^−6^ mol/L) for 30 min followed by E_2_ (10^−7^ mol/L) for 4 h. Then, cells were crosslinked, lysed, and sonicated by Sonicator JY92-II (SCIENTZ, Ningbo, China). The lysate was pre-absorbed with protein A/G-agarose (Santa Cruz Biotechnology, CA, USA), then incubated with indicated antibodies. The immunocomplexes were precipitated by protein A/G-agarose and the crosslinked DNA samples were amplified by PCR. The primers sequences corresponding to position +59 to +340 of the *vav*1 relative to TSS were: sense, 5′- CTGCGAGGGTGCACGG-3′; and antisense, 5′- GTCTCCAACCCTCAGCACAC -3′. The primers for position -232 to +71 were: sense, 5′- GAGGAAGCTCACCCATCTCA -3′; and antisense, 5′- TGCACCCTCGCAGCCTCCA -3′.

### Knockdown of Vav1 expression by lentivirus-based transduction

The lentiviral plasmids were constructed as described [32]. The shRNA sequence targeting Vav1 or control RNA of scrambled sequence were cloned into pLKO.1-TRC vector (Addgene, http:/www.addgene.org/), respectively. The lentivirus particles were generated according to the standard protocol [33]. Briefly, HEK293T were co-transfected with the vectors containing shRNAs together with vectors pCMV-VSV-G, pMDLg/pRRE, and pRSV-REV. At 48 h post-transfection, the supernatants were harvested, and the viral particles were collected to infect T47D cells at 37°C for 18 h. The transduced cells were selected by 0.5 µg/mL puromycin for 7 days.

### Cell cycle analysis

Cells were synchronized to G0/G1 phase by cultured in serum-free medium in the presence of Tamoxifen [34]. After 36 h of treatment with E_2_ (10^−7^ mol/L) or DMSO as control, cells were collected and fixed with ice-cold 70% ethanol, then incubated with 100 µg/mL RNase A (Transgene Biotech, Beijing, China) for 30 min. Cells were then stained with 50 µg/mL propidium iodide (PI) (Sigma, MO, USA) in the dark for 30 min, and subjected to flow cytometer analysis (Calibur, NJ, USA). The DNA contents and cell numbers were plotted using Cell Quest software (Becton Dickinson, NJ, USA).

### Western Blot analysis

Cells (1×10^6^ per sample) were harvested and lysed in RIPA buffer (50 mmol/L Tris-HCl, pH 7.5, 150 mmol/L NaCl, 5 mmol/L EDTA, 1% Triton X-100, 1 mmol/L PMSF, 1 mmol/L NaF, 1 µg/mL leupeptin, 1 µg/mL pepstatin, and 1 mmol/L Na_3_VO_4_). Protein concentration was measured by Bradford assay. Equal protein amounts of cell lysates were separated by 7.5% or 12% SDS-polyacrylamide gel electrophoresis (SDS-PAGE), transferred to PVDF membranes, and blotted with indicated antibodies. The density of each band was quantitated by Quantity One software (Bio-Rad, version 4.4.0, CA, USA).

### Co-immunoprecipitation (Co-IP)

T47D cells were cultured in RPMI 1640 medium for 24 h before adding E_2_ (10^−7^ mol/L) or DMSO for 4 h. Then cells were harvested and lysed in RIPA buffer. 1.5 mg of lysate was precleared with protein A/G-agarose beads and subsequently added in 4 µg of indicated antibodies overnight at 4°C. The immunocomplexes were precipitated by protein A/G-agarose, washed 3 times with RIPA buffer, subjected to SDS-PAGE, and analyzed by Western Blot.

### Statistics analysis

Graphical data values are presented as mean values of triplicate experiments ± standard deviations. Each experiment was carried out independently for at least 3 times, and unpaired student T tests were performed. The statistical significance was set at P<0.05 (marked with *) and P<0.01 (marked with **).

## Results

### The aberrant expression of Vav1 in human breast cancer tissue and cell lines

**Figure 1:**
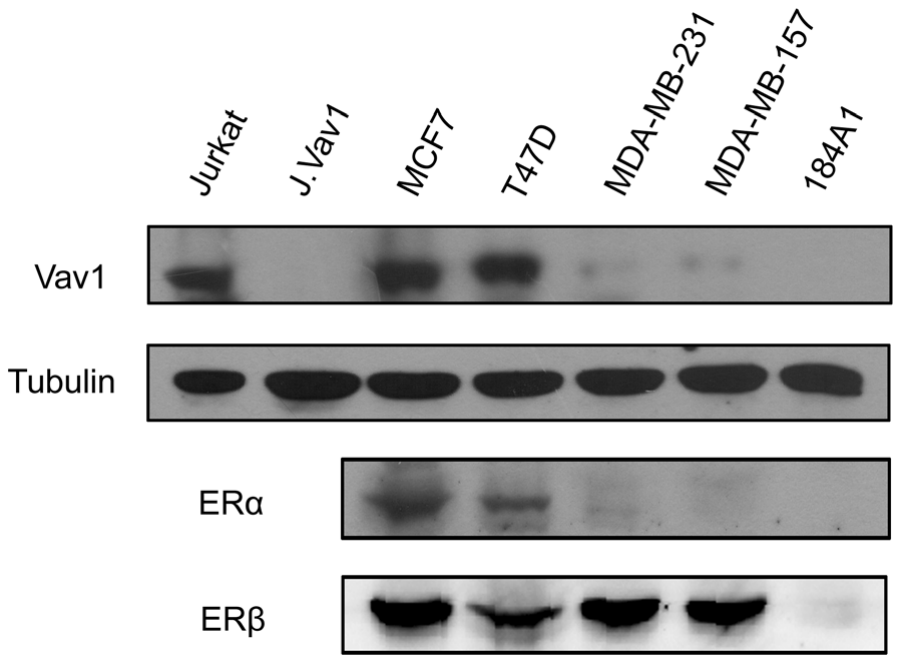
Expression of Vav1 in human breast cancer cell lines. Western Blot analysis of Vav1 and estrogen receptors expression in human breast cancer cell lines (ER positive: MCF7 and T47D; ER negative: MDA-MB-231 and MDA-MB-157) and immortalized breast epithelial line 184A1. The blot with anti-α-tubulin antibody served as a loading control (lower panel). Jurkat cells and its derived *vav*1-null cells (J.Vav1) were used as positive and negative controls for Vav1.

It was reported previously that Vav1 was detected in ER-positive breast cancer tissue by immunohistochemistry [27]. Here we examined the expression of Vav1 in human breast cancer cell lines (Fig. 1), using Vav1 abundant Jurkat cells as positive control and its derived *vav*1-null cells (J.Vav1) as negative control (Fig. 1, left two lanes). As shown in Figure 1, Vav1 expression appeared high in the two ER positive cell lines, MCF7 and T47D cells, whereas it was barely detectable in MDA-MB-231 and MDA-MB-157, and not detected in the immortalized breast epithelial line 184A1 cells. The estrogen receptors expression of the cell lines was also detected.

### Estrogen enhances Vav1 expression through ERs in MCF7 and T47D cell lines

**Figure 2:**
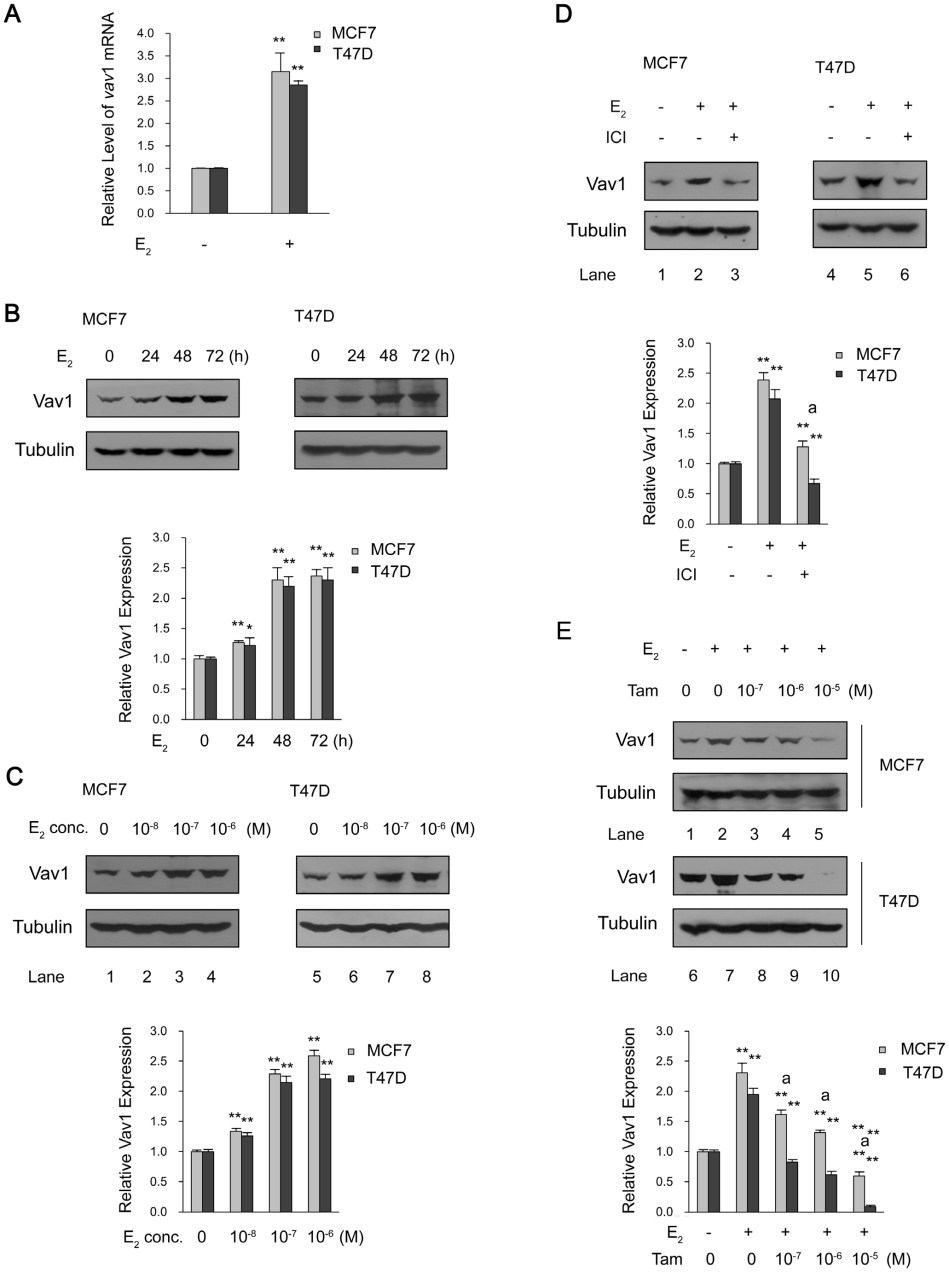
ER-mediated Vav1 expression. (A) MCF7 and T47D cells were treated with E_2_ (10^−7^ mol/L) or DMSO for 24 h. The relative level of *vav*1 mRNA was determined by qRT-PCR and was presented by the ratio of *vav*1 mRNA of E_2_-treated samples to that of DMSO-treated control samples and presented as *y*-axis. The data represented the mean value±S.D. of three independent experiments. (B and C) MCF7 and T47D cells were exposed to E_2_ (10^-7^ mol/L) for 0 to 72 h (B), or increasing concentration of E_2_ for 48 h (C). (D and E) MCF7 and T47D cells were pre-treated with ICI 182,780 (4×10^−7^ mol/L) (D) or increasing concentration of Tamoxifen for 30 min (E) before adding E_2_ (10^−7^ mol/L) for 48 h. The DMSO treatment was used as a solvent control. The Vav1 expression in above treated samples was analyzed by Western Blot with anti-Vav1 antibody, with tubulin as protein loading control. The bar chart below each example blot represents the normalized protein level of Vav1 to Tubulin of three independent experiments. The DMSO treatment was set as 1 to indicate the basal level of Vav1 expression. “**” indicates P<0.01 versus DMSO treatment and “a**” indicates P<0.01 versus E_2_ treatment by unpaired student T test.

A correlation has been observed between the progression of breast cancer and the exposure to estrogen, which modulates the transcription of many genes by binding and activating ERs [2]–[5]. From the results of tissue immunohistochemistry [27] and cell lines Western blot (Fig. 1), higher Vav1 expression was visualized in ER positive breast tumors or cells than that in ER negative samples or cells. We speculated that estrogen-ER was involved in the control of *vav*1 gene expression. Two ER-positive cell lines, MCF7 and T47D, were tested for Vav1 expression in the presence or absence of 17β-estradiol (E_2_). After treatment with 10^−7^ mol/L of E_2_ or DMSO as control, the mRNA transcript of *vav*1 was measured by qRT-PCR. As shown in Figure 2A, E_2_ induced an increase in *vav*1 mRNA expression by 3.15-fold in MCF7 and 2.86-fold in T47D in reference to the DMSO control (P<0.01), suggesting that E_2_ enhanced the transcription of *vav*1 gene.

We further explored the effects of time and concentration of E_2_ on Vav1 expression. MCF7 and T47D cells were exposed to E_2_ (10^−7^ mol/L) for different time points, or to the indicated concentration of E_2_, respectively. As shown in Figure 2B, the E_2_-induced Vav1 expression increased to about 2.2 fold at 48 h (P<0.01) and plateaued at 72 h in both cell lines. The expression of Vav1 reached to nearly maximum at 10^−7^ mol/L of E_2_, as only limited increases of Vav1 from 10^−7^ mol/L to 10^−6^ mol/L were observed, namely from 2.29 to 2.59 fold for MCF7 and 2.15 to 2.21 fold for T47D, respectively (Fig. 2C). The above data indicated that the induction of Vav1 expression is dependent on the time and dose of the ER ligand treatment.

Given that ICI 182,780 and Tamoxifen have been applied in endocrine therapy for ER-positive breast cancer due to their inhibitory effects on ER activation [35]–[37], we used these drugs to address the role of ER in the estrogen regulation of Vav1. As shown in Figure 2D, E_2_ alone induced Vav1 protein expression by 2.39-fold and 2.08-fold in MCF7 and T47D cells (Fig. 2D, Lane 2 and 5, P<0.01), respectively. However, this expression was suppressed by ICI 182,780 to the basal level (Fig. 2D, Lane 3 and 6, P<0.01). Similarly, Tamoxifen counteracted E_2_ and inhibited Vav1 expression to the level below the baseline at high concentrations (Fig. 2E, lanes 3–5 and 8–10, P<0.01). These data suggested that Vav1 expression was not only dependent on the time and dose of estrogen, but also required the activation of ERs in breast cancer cell lines.

### ER increases the promoter activity of *vav*1 gene

**Figure 3:**
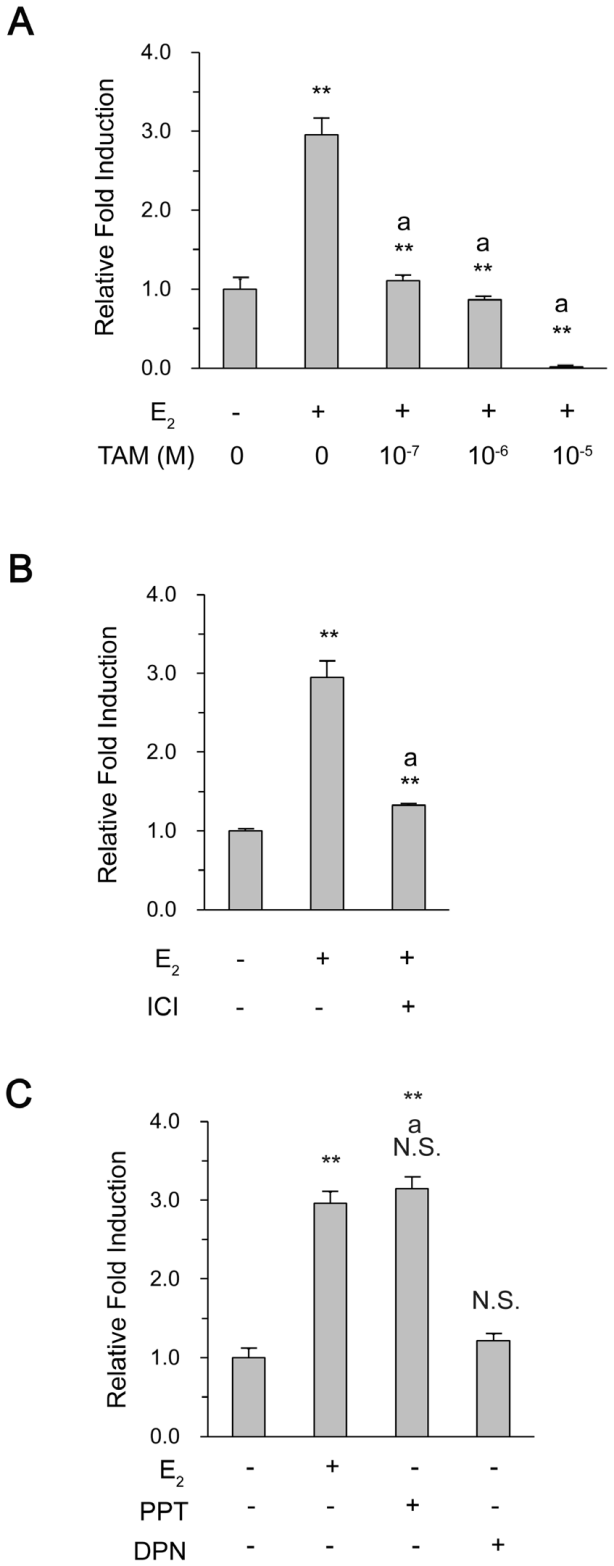
Activation of *vav*1 promoter by ERα. MCF7 cells were transfected with the *vav*1 luciferase reporter gene, and cultured in RPMI 1640 medium for 24 h. The cells were pre-treated with increasing concentration of Tamoxifen (A) or ICI 182 782 at 4×10^−7^ mol/L (B) for 30 min, followed by E_2_ treatment for another 48 h. Or the cells were treated with E_2_ (10^−7^ mol/L), PPT (10^−7^ mol/L), or DPN (10^−7^ mol/L) for 48 h (C). The DMSO treatment served as solvent control. The induction fold of luciferase activity was measured as described in Methods, and plotted as the ratio to that of the control in *y*-axis. The data represented the mean±S.D. of three independent experiments. “**” and “N.S.” indicate P<0.01 and P>0.05, respectively, versus DMSO treatment by unpaired student T test; “a**” and “aN.S.” indicate P<0.01 and P>0.05, respectively, versus E_2_ treatment.

As E_2_-ER efficiently enhanced Vav1 protein as well as mRNA expression, we predicted that ERs would function as a transcriptional activator for *vav*1 gene promoter. The minimal regulatory sequences of *vav*1 proximal promoter region, which covered nucleotide (nt) −287 to +301 relative to Transcription Start Site (TSS), was constructed in plasmid pGL3 as described [30], and the resulting plasmid was named pVav1-Luc. In agreement with Vav1 protein expression in Figure 2, E_2_ treatment induced a maximal activation of *vav*1 promoter, nearly 3 fold above the control (DMSO treatment) (Fig. 3A, P<0.01). The presence of Tamoxifen decreased the *vav*1 promoter activity to the basal level, and the promoter activity exhibited a negative correlation with the increasing concentration of the drug (Fig. 3A). Similarly, ICI 182,780 suppressed the reporter gene by 1.33 fold (Fig. 3B, P<0.01 versus E_2_ treatment). The above results suggested that ERs were involved in the activation of *vav*1 promoter activity, and thus the transcriptional activation of *vav*1 gene.

### The transcription of *vav*1 gene is mediated by the α form of ER

By far, two ER forms, ERα and ERβ, were known to play significant roles in diverse tissues, and both were detected in MCF7 and T47D cell lines (Fig. 1). We attempted to identify the one which modulated *vav*1 transcription by using chemical agonist specific for ERα (PPT), or ERβ (DPN). The cells transfected with pVav1-Luc were treated with DMSO, E_2_, PPT, or DPN, respectively. The luciferase activity was measured and presented as fold of induction to that treated by DMSO. As shown in Figure 3C, E_2_ and PPT induced the promoter activation to the same extent (∼3-fold, P<0.01 versus DMSO treatment and N.S. between E_2_ and PPT groups), whereas, the ERβ agonist DPN did not show any significant induction above the control (DMSO). Thus, only ERα, but not ERβ, was responsible for E_2_-induced *vav*1 transcription.

### ERα is involved in the complex associated with *vav*1 promoter

**Figure 4:**
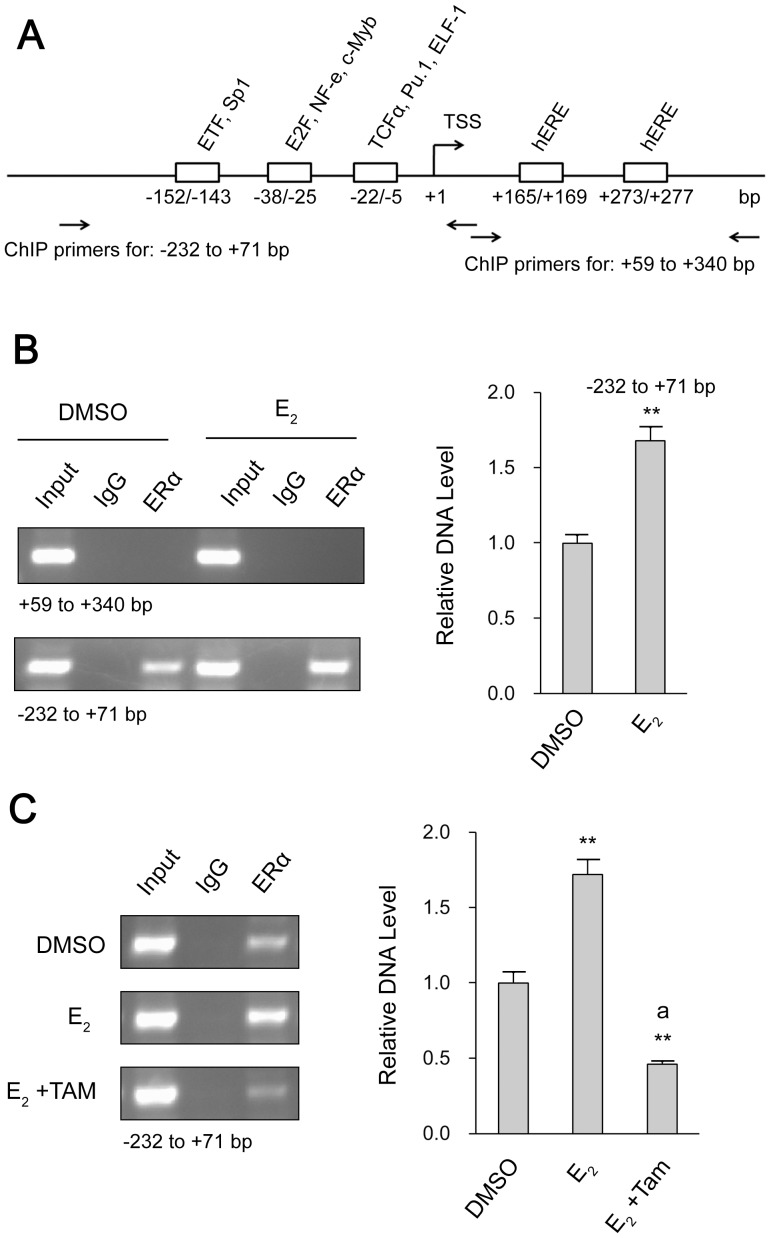
ChIP analysis of ERα with the *vav*1 promoter DNA. (A) Schematic representation of the *vav*1 proximal promoter region. The predicted transcription factors and hERE sites were framed by boxes. Horizontal arrows indicated the primers used for PCR in ChIP assays. TSS: transcription start site. hERE: half estrogen response element (ERE). (B) T47D cells were treated with E_2_ (10^−7^ mol/L) or DMSO (solvent control) for 4 h and ChIP analysis was performed with anti-ERα antibody or control IgG. Two sets of primers specific for +59 to +340 region containing hERE sites (upper panel) or the −232 to +71 region of the *vav*1 promoter (lower panel) were used in PCR. The PCR products were detected by agarose gel electrophoresis. The input represented the DNA in crude cell extract before the immunoprecipitation. (C) T47D Cells were treated with the reagents as indicated in the left side, and ChIP assay were carried on using primers specific for −232 to +71 of *vav*1 promoter. The PCR products were resolved by agarose gel electrophoresis. The bar chart beside each example blot represents the normalized DNA level of −232 to +71 to Input of three independent experiments. “**” indicates P<0.01 versus DMSO treatment and “a**” indicates P<0.01 versus E_2_ treatment by unpaired student T test.

The involvement of ERα in estrogen-induced *vav*1 transcription led us to examine the *vav*1 proximal promoter for conserved ERE sequence *in silico* by rVista2.0 (http://rvista.dcode.org/) and TRANSFAC (http://www.cbrc.jp/htbin/nph-tfsearch). However, the search result revealed no perfect ERE at the *vav*1 promoter region, rather, there were two half-ERE sites (hERE) located at the positions +165 to +169 bp and +273 to +277 bp to TSS, respectively (Fig. 4A). As previously reported, ERE-like sequence, such as two half ERE sites, can bind with estrogen activated ER even though they were separated by hundreds of base pairs [9] [38]. Thus we set to verify if ERα bound to the hERE sites at *vav*1 promoter by ChIP analysis. The primers corresponding to the region spanning the two hERE sites (+59 to +340) were designed accordingly. As shown in Figure 4B upper panel, the sample prior to immunoprecipitation (Input) exhibited a positive hERE region, whereas was detected negative in the post-immunoprecipitated sample (ERα), indicating that ERα did not interact with the hERE sites. Unexpectedly, the region −232 to +71 was found in association with ERα (Fig. 4B, lower panel, third lane from the left), though there was no consensus binding site for ER. Furthermore the recruitment of ERα was increased by ∼1.7 fold upon E_2_ treatment (Fig. 4B, lower panel, sixth lane from the left, P<0.01), and reduced by Tamoxifen treatment (Fig. 4C, P<0.01 versus DMSO and E_2_ treatment). The above results demonstrated that ERα was involved in the transcriptional activation of *vav*1 gene by association with the promoter region other than the hERE sites, implying an indirect binding of ERα to the promoter region, perhaps through other transcription factors.

### ERα associates with −38 to −5 region at *vav*1 promoter via other transcription factors

**Figure 5:**
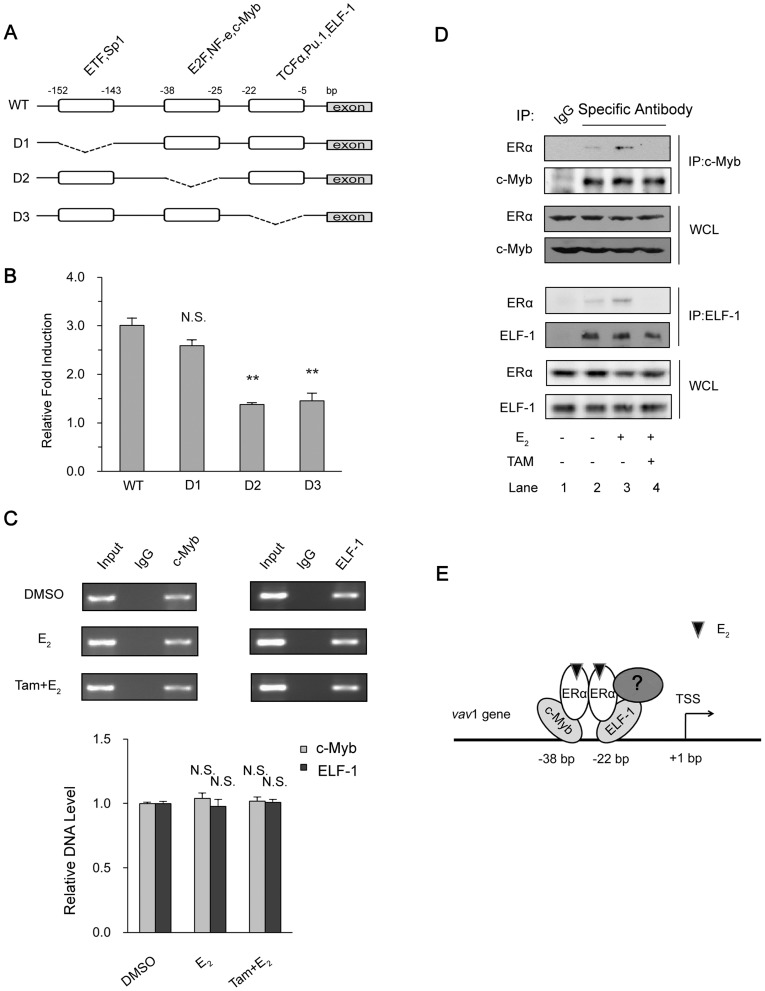
Analysis of transcription factors involved in ERα-activated *vav*1 promoter. (A) Depicted deletion mutations in *vav*1 promoter reporter gene. The deleted regions were indicated by break lines. (B) MCF7 cells were transfected with plasmids containing luciferase under WT or mutated *vav*1 promoters (D1, D2, and D3) and then treated with E_2_ (10^−7^ mol/L) or DMSO for 48 h. The relative fold induction of each group was calculated as the ratio of the luciferase activity induced by E_2_ to that induced by DMSO, respectively, and plotted as *y*-axis, and the deletion mutants were presented as in *x*-axis. All the data represented the mean±S.D. of three independent experiments. “N.S.” and “**” indicates P>0.05 and P<0.01, respectively, versus D1 group by unpaired student T test. (C) ChIP assay. T47D cells were treated with E_2_ (10^−7^ mol/L) for 4 h or pre-treated with Tamoxifen (10^−6^ mol/L) for 30 min before treating with E_2_ (10^−7^ mol/L) for 4 h. The immunoprecipitation was performed using antibodies against c-Myb (left panels) or ELF-1 (right panels), with preimmune IgG as control. The precipitated DNAs were analyzed by PCR with the primers corresponding to position −232 to +71 of *vav*1 promoter. The bar chart below the example blot represents the normalized DNA level of −232 to +71 to Input of three independent experiments. “N.S.” indicates P>0.05 versus DMSO treatment by unpaired student T test. (D) The association of ERα with c-Myb and ELF-1. T47D cells were treated with E_2_ (10^−7^ mol/L) or DMSO for 4 h or pretreated with Tamoxifen (10^−6^ mol/L) for 30 min in prior to E_2_ treatment and lysed. Antibodies against c-Myb (upper panels) and ELF-1 (lower panels) or control IgG (Lane 1) were used to immunoprecipitate protein complex, which were then resolved by Western Blot with indicated antibodies. (E) The proposed model for ERα modulating the *vav*1 promoter activity. E_2_-activated ERα interacts with *vav*1 promoter via interacting with transcription factors such as c-Myb, ELF-1, or perhaps other unknown coregulators (labeled “?”) to promote the *vav*1 transcription.

The above results indicated that ERα was in complex with the 5′ region of *vav*1 gene promoter. Several transcription factors were predicted to bind at the 5′ minimal regulatory region of the human *vav*1 gene, including ETF, Sp1, E2F, NF-e, c-Myb, TCFα, PU.1, and ELF-1 [30]. We therefore attempted to locate the regions that respond to estrogen. The wild type *vav*1 promoter (WT) and the truncated mutants (D1, D2, D3) that lack the predicted transcription factor binding sites were depicted in Figure 5A, and the reporter plasmids were constructed [30]. As shown in Figure 5B, the wild type promoter activity was elevated to 3 fold by E_2_. The deletion mutant D1 that lacks region -(143∼152) exhibited similar extent of induction (2.6 fold), implying that the region −(143∼152) was dispensable in E_2_-induced *vav*1 expression. In contrast, the E_2_ induction of truncated promoters D2 and D3 was severely suppressed to less than 1.5 fold (P<0.01), indicating that these two regions, −(25∼38) and −(5∼22), were required for E_2_-induced *vav*1 transcription. As these regions were reported to possess putative binding sites for transcription factors E2F/NF-e/c-Myb at -(25∼38) and TCFα/PU.1/ELF-1 at −(5∼22), respectively, ERα may associate with certain transcription factors within these regions. As reported previously, c-Myb affects *vav*1 transcription in lung cancer cells [30] and is also involved in the E_2_-ER regulated gene expression in breast cancer cells [39]. Meanwhile, another breast cancer related transcription factor, ELF-1, is identified to interact with the promoter of *vav*1 (Genome browser, http://genome.ucsc.edu/) [40]. Firstly, to confirm the binding of these two transcription factors to *vav*1 promoter, the ChIP analysis was performed. As shown in Figure 5C, both c-Myb and ELF-1 presented positively in complex with the *vav*1 promoter (Fig. 5C, upper two panels), and the presence of E_2_ or Tamoxifen had no effects on the complex (Fig. 5C, bottom panel).

Next we investigated whether ERα associated with c-Myb and/or ELF-1 to form the transcriptional complex, and if the complex formation was E_2_-dependent. By co-immunoprecipitation analyses shown in Figure 5D, detectable amount of ERα was pulled down with anti-c-Myb (upper panels) or anti-ELF-1(lower panels) antibodies, respectively, in comparison with control IgG (Fig. 5D, lane 2 versus lane 1). And the amount of co-immunoprecipitated ERα increased with the presence of E_2_ (Lane 3 in both upper and lower panels), indicating the E_2_-activated ERα associated with the transcription factors c-Myb and ELF-1. In the presence of Tamoxifen, the co-immunoprecipitated ERα was barely detectable (Fig. 5D, lane 4), further suggesting that it was the activated form of ERα that bound to these transcription factors.

Given that the existing interaction of c-Myb/ELF-1 with *vav*1 promoter was constitutive (Fig. 5C), and ERα association with -232 to +71 region was E_2_ inducible (Fig. 4C), we proposed that the E_2_ dependent activation of *vav*1 promoter was achieved by the association of resident transcription factors such as c-Myb and ELF-1, with E_2_-activated ERα. As the model shown in Figure 5E, E_2_ induced activation of ERα, which, instead of binding directly to *vav*1 promoter, interacted with the existing transcription factors to control the *vav*l transcription. Of course, our results did not exclude the involvement of other regulators in the complex. Nevertheless, the recruitment of ERα and the formation of the transcriptional complex enhanced the transcription of *vav*1 gene.

### The expression of Vav1 promotes cell cycle progression in breast cancer cells

**Figure 6:**
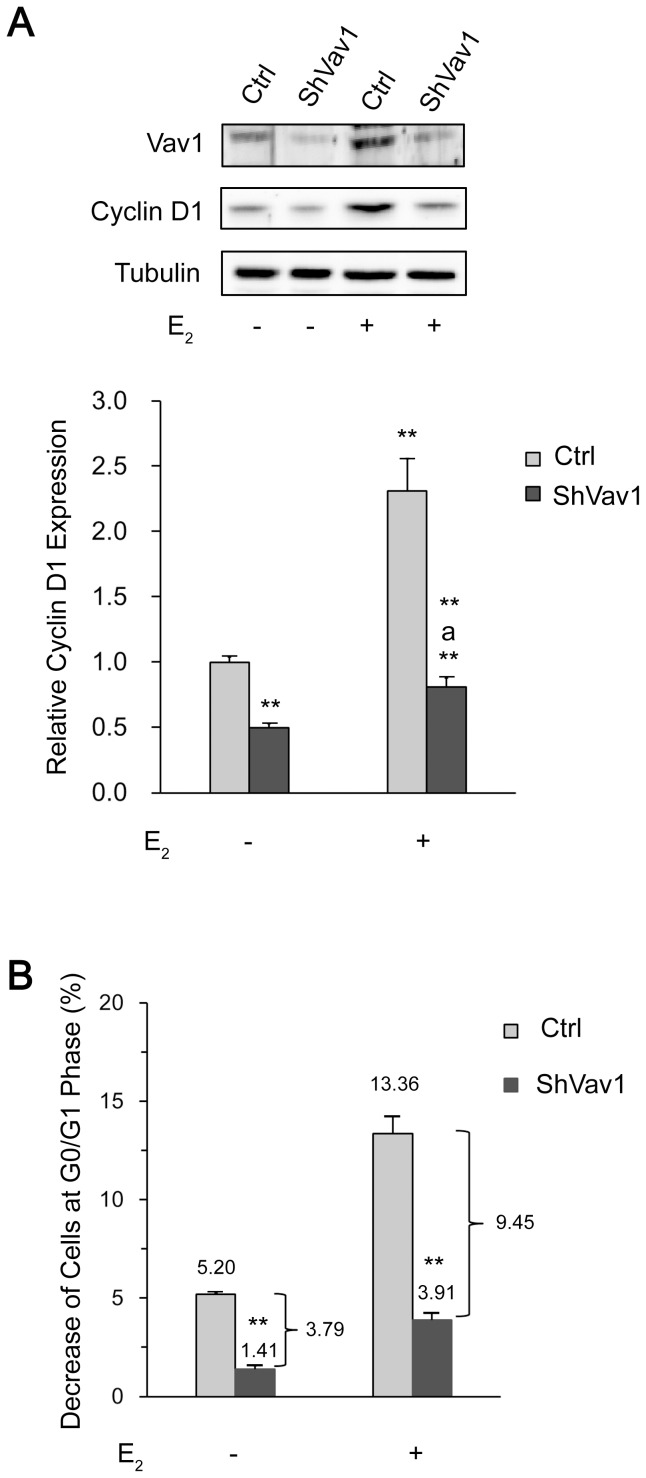
Effect of Vav1 in cell cycle progression. (A) T47D cells were infected with lentivirus particles that expressed Vav1-specific shRNA or shRNA with a scramble sequence (served as a control). The homogenous cells were first synchronized to G0/G1 phase and then treated with DMSO as control or E_2_ (10^−7^ mol/L) for 36 h before harvest. The expression of Vav1 and Cyclin D1 were analyzed by Western Blot with indicated antibodies respectively. The density of the bands was quantitated by Quantity One software (Bio-Rad, version 4.4.0, CA, USA). The bar chart represents the normalized protein level of Cyclin D1 to tubulin of three independent experiments. “**” indicates P<0.01 versus DMSO treatment of Control, and “a**” indicates P<0.01 versus E_2_ treatment of Control by unpaired student T test. (B) Another aliquots of above cells were stained by PI and analyzed by flow cytometer for DNA contents. The decrease in the percentage of cells in G0/G1-phase before and after the treatment by DMSO or E_2_ was calculated and plotted as y-axis and labeled on the bar graph. The differences between two T47D-Ctrl and T47D-ShVav1 cells were also marked. The data represented the mean ±S.D. of three independent experiments. “**” indicates P<0.01 versus corresponding control group by unpaired student T test.

E_2_ is identified as a causative factor of breast cancers and well-characterized to induce cell growth in ER-positive breast tumors [34]. As Vav1 is also involved in cell proliferation in lung cancer and pancreatic cancer cells [22], [23], we speculated that Vav1 participated in cell cycle progression of breast cancer cells under the control of E_2_. Stable cell lines, T47D-ShVav1 expressing short hairpin RNA for Vav1, and T47D-Ctrl expressing a scrambled sequence, were established by lentivirus-based transduction. The expression of Vav1 in these cell lines was verified as shown in Figure 6A, and the level of Vav1 expressed in shVav1 cells was reduced (Fig. 6A, top panel, the left two lanes). The E_2_ treatment elevated the Vav1 expression proportionally (Fig. 6A, top panel, the right two lanes). The expression of Cyclin D1 was also determined as a commonly recognized factor for cell cycle progression (Fig. 6A, middle panel). In the absence of E_2_, shVav1 reduced Cyclin D1 by 50% (P<0.01) in comparison with the control (left lane). E_2_ induced a significant 2.31-fold increase of Cyclin D1 (Fig. 6A, third lane from the left, P<0.01) in coordination with Vav1, and that was reduced by the shRNA knockdown of Vav1 (P<0.01 versus E_2_ treatment of Control).

Furthermore, we examined the effects of Vav1 on the cell proliferation by flow cytometry analysis. The shRNA-transduced breast cancer cells were synchronized to G0/G1 phase and the G0/G1 arrest was released by E_2_ or DMSO treatment as described in Methods. The decreased percentage of cells in G0/G1 phase represented the cells progressing to cell cycle. As in Figure 6B, in the absence of E_2_, 5.20% of T47D-Ctrl cells and 1.41% of T47D-ShVav1 cells were released from the G0/G1 checkpoint (Fig. 6B, left bars), respectively. With E_2_ treatment, 13.36% of the T47D-Ctrl cells reentered cell cycle, whereas only 3.91% of T47D-ShVav1 cells were progressed (Fig. 6B, right bars). Thus, knockdown of Vav1 resulted in a 3.79% reduction in DMSO group and a 9.45% reduction in E_2_ group. The differences of the percentage of proliferating cells with shVav1 or not reckoned for the relative amount of Cyclin D1 of those cells (Fig. 6A), and revealed that knockdown of Vav1 decreased E_2_-induced cell proliferation. In addition, overexpression of Vav1 in T47D cells also exhibited an enhanced Cyclin D1 by 2.5 fold (Fig. S1A), and a higher cell growth rate was also shown by WST-1 analysis (Fig. S1B). The implications from the above experiments are 2-fold, 1) Vav1 expression level significantly influences the cell cycle progression and cell proliferation; and 2) The amount of Vav1 protein contributes the E_2_-upregulated cell proliferation.

## Discussion

Vav1 has been recognized as a hematopoietic-specific protein and plays important roles in T cell activation. The non-hematopoietic expression of Vav1 has been reported recently in association with several human malignancies, including pancreatic ductal adenocarcinomas [23], lung cancer [22], neuroblastoma [21], melanoma [41], and breast cancer [24]–[27]. Our present study revealed that Vav1 protein expression was observed in some breast cancer cell lines by Western Blot, especially in ER+ cell lines, though with discrepancy in MCF-7 cell line [27]. It was mutually believed that such discrepancy may result from the resources and passage numbers of the cell line (personal communication with Dr. Katzav). Nevertheless, Vav1 was aberrantly expressed in breast cancer tissue and cell lines.

Several studies are attempted to explore the mechanisms involved in non-hematopoietic expression of Vav1. Epigenetic indication of *vav*1 expression is proposed. For example, the demethylation of the *vav*1 gene promoter is detected in primary pancreatic adenocarcinomas [23]; methylation of CpG in 5′-regulatory sequences of the *vav*1 promoter is addressed in lung cancer cells [30]; and degradation of Vav1 through Cbl ubiquitination is proposed in breast cancer cells [27]. Given a positive correlation between Vav1 and ER expression in breast cancer tissue [27], we were motivated to explore the Vav1 expression along the E_2_-ER axis. Our data unveiled the transcriptional control of the *vav*1 gene under the estrogen-ER pathway, and the required isoform, ERα, was the dominant form in breast tissue [42].

A plethora of genes are reported to be modulated by estrogen-ER pathway via estrogen response elements. We analyzed the *vav*1 promoter sequence *in silico*, and identified two half ERE sites. However, it was not supported by the ChIP analysis, as these sequences did not bind and recruit ERα (Fig. 4B). Rather, promoter sequences stretching from −232 to +71 bp to TSS associated with ERα indirectly. Further, two regions in the *vav*1 promoter were found indispensable for E_2_-induced reporter activity, which contained the binding sites for c-Myb and ELF-1, respectively. Accumulating evidence does support this scenario that ER indirectly activates gene transcription via binding to other DNA-bound transcription factors [10]–[12], . For example, ERα complexes with c-Myc to mediate the expression of Noxa in breast cancer cells [12]. The association of ER with Sp1 is also reported to modulate c-fos expression [11]. In our data, ERα, by interacting with c-Myb and ELF-1, conferred estrogen responsiveness to *vav*1 gene (Fig. 5E). Indeed, c-Myb and ELF-1 possess the leucine-rich motif: LXXLL, which can be recognized by ERα [43]. Of course, other coregulators involved in complex with ERα remain to be identified.

Two activation functions (AFs) mediate the transcriptional activation of ER, the N-terminal AF-1, and the AF-2 in ligand binding domain (LBD) [44]. The biological ligand, E_2_, binds to LBD and induces its conformational change to trigger the activity of AF-2 which can be recognized by coactivators [43]. Previous studies suggest that Tamoxifen, the non-steroidal type I ER antagonist, induces a different conformation from that induced by E_2_, and thus blocks the binding of coactivators and inhibits AF-2 activity [45], [46]. By contrast, ICI 182,780, a steroidal type II ER antagonist, binds competitively to the E_2_ binding site, blocks ER activation, and leads to a rapid degradation of ER [35]. As both Tamoxifen and ICI 182,780 eliminated E_2_-induced *vav*1 expression (Fig. 3), and the Co-IP analysis revealed that the association of ERα with the two cofactors was disrupted by Tamoxifen (Fig. 5D), we speculated that the correct conformation of ERα is required for its complex with the DNA-bound c-Myb and ELF-1 to access *vav*1 promoter, thus control *vav*1 transcription.

Elevated expression of Vav1 has been demonstrated to affect cell proliferation in lung cancer and pancreatic cancer cells [22], [23]. As a GDP/GTP exchange factor, Vav1 is also involved in CXCL12-promoted invasion of melanoma cells [41]. In this study, we observed that the aberrant expression of Vav1 correlated well with the production of Cyclin D1, a critical mediator of estrogen-stimulated cell cycle progression [47], [48], thus contributing to the proliferation of breast cancer cells (Fig. 6, Fig. S1). We could not rule out other mechanisms that Vav1 played in corresponding to the growth and development cancer, as Vav1 might also protect cells from apoptosis by enhancing anti-apoptotic Bcl2 transcription that we reported in leukemia cells [49].

In summary, our data revealed that E_2_ promoted the expression of *vav*1 in a dose- and time-dependent manner in ER-positive breast cancer cells. E_2_-ER indirectly enhanced *vav*1 transcription, perhaps via the interactions with other transcription factors such as c-Myb and ELF-1. The E_2_-induced Vav1 level in breast cancer cells was in favor of promoting Cyclin D1 expression and accelerating the cell proliferation, and Vav1 might partially contribute to the pathogenesis and prognosis of breast cancer. This study emphasized the involvement of Vav1 ectopic expression in ER positive breast cancer cells, which reinforced the hypothesis that Vav1 could exert its oncogenic role in human breast cancer.

## Supporting Information

Figure S1 **Effect of Vav1 overexpression on cell proliferation**. T47D cells were transduced with lentivirus particles encoding Vav1 (pCDH-Vav1) or the control vector backbone (pCDH). The expression of Vav1 and Cyclin D1 were analyzed by Western Blot and cell proliferation was determined by WST-1 proliferation assay in these cells.
(TIF)

Materials and Methods S1 **Overexpression of Vav1 by lentivirus-based transduction and WST-1 cell proliferation assay.**
(DOC)
