# Supplementary figures and images for: Estrogen Induces Vav1 Expression in Human Breast Cancer Cells
Source: PLoS One. 2014 Jun 6;9(6):e99052. doi: 10.1371/journal.pone.0099052 (PMC4048212; doi:10.1371/journal.pone.0099052)

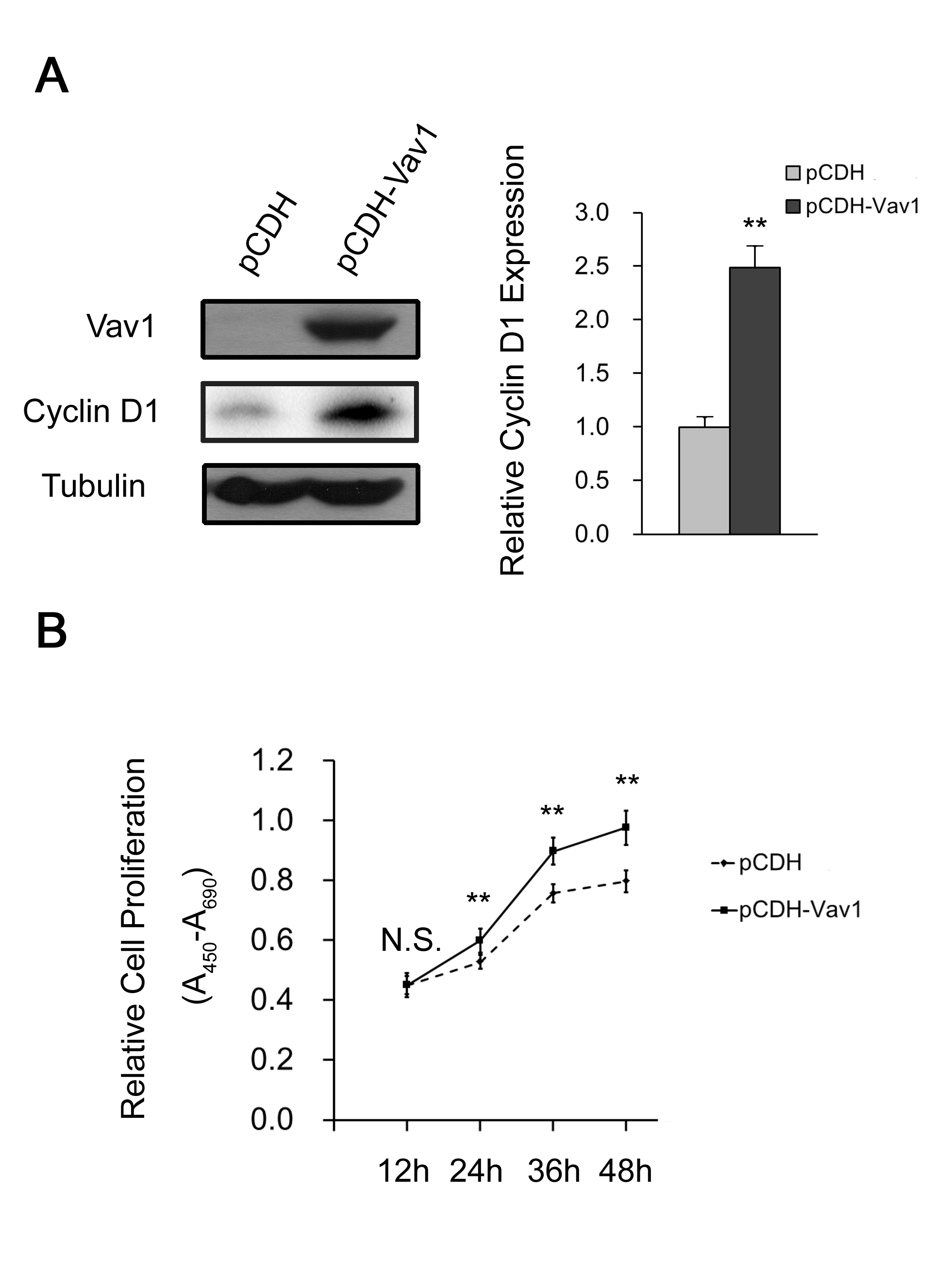

Supplement: Figure S1 — Effect of Vav1 overexpression on cell proliferation. T47D cells were transduced with lentivirus particles encoding Vav1 (pCDH-Vav1) or the control vector backbone (pCDH). The expression of Vav1 and Cyclin D1 were analyzed by Western Blot and cell proliferation was determined by WST-1 proliferation assay in these cells. (TIF) [file pone.0099052.s001.tif]
