# Supplementary material for: Estrogen Induces Vav1 Expression in Human Breast Cancer Cells
Source: PLoS One. 2014 Jun 6;9(6):e99052. doi: 10.1371/journal.pone.0099052 (PMC4048212; doi:10.1371/journal.pone.0099052)
Supplement: Materials and Methods S1 — Overexpression of Vav1 by lentivirus-based transduction and WST-1 cell proliferation assay. (DOC) [file pone.0099052.s002.doc]

**Overexpression of Vav1 by lentivirus-based transduction**

The lentiviral plasmids were constructed as described . Vav1 cDNA fragment was subcloned into pCDH-CMV-MCS-EF1-puro vector. The lentivirus particles were generated following a standard protocol as instructed . Briefly, HEK293T were co-transfected with the coding DNA of Vav1 together with packaging vectors pCMV-VSV-G, pMDLg/pRRE, and pRSV-REV. At 48 h post-transfection, the supernatant were harvested, and the viral particles were collected to infect breast cancer cells T47D at 37°C for 18 h. The transduced cells were selected by 0.5 μg/mL puromycin for 7 days before analyses.

**WST-1 cell proliferation assay**

T47D cells stably expressing Vav1 or vector were plated at a concentration of 1 x 10^3^ cells per well (100 μL) in 96-well plates. After different period of time, 10 μL of WST-1 reagent (Beyotime Institute of Biotechnology, Beijing, China) was added into each well and the absorbance was measured at 450 nm and 690 nm after 2.5 h of incubation using Model 680 Microplate Reader (Bio-Rad). The difference of absorbance at 450 nm and 690 nm was calculated.

1. Li SY, Du MJ, Wan YJ, Lan B, Liu YH, et al. (2013) The N-terminal 20-amino acid region of guanine nucleotide exchange factor Vav1 plays a distinguished role in T cell receptor-mediated calcium signaling. J Biol Chem 288: 3777-3785.

2. Wang X, McManus M (2009) Lentivirus production. J Vis Exp.
